# New-Onset Atrial Fibrillation and Multiple Systemic Emboli in a COVID-19 Patient

**DOI:** 10.7759/cureus.12917

**Published:** 2021-01-26

**Authors:** Omar Al-Abbas, Alfarooq Alshaikhli, Hashed A Amran

**Affiliations:** 1 Family Medicine Residency Program, Medical Education, Hamad Medical Corporation, Doha, QAT; 2 Internal Medicine, Beaumont Health, Michigan, USA

**Keywords:** covid-19, sars-corona virus 2, electrocardiogram, atrial fibrillation, palpitation, direct acting oral anticoagulant, systemic embolisms

## Abstract

Severe acute respiratory syndrome coronavirus 2 (SARS-CoV-2) infection was declared a pandemic by the World Health Organization in March 2020. Initially, infected patients presented with fever, nonproductive cough, dyspnea, myalgia, shortness of breath, and radiographic evidence of pneumonia. However, others presented with atypical cardiac manifestation. As this disease is new, the full picture of the disease presentation is not fully understood. Since December 2019, many morbidities related to coronavirus disease-2019 (COVID-19) were documented, including vascular complications like venous thromboembolism (VTE), pulmonary embolism (PE), and deep vein thrombosis (DVT) in acutely ill COVID-19 patients. Hereby, we are writing a case of a patient with COVID-19 infection suffering from new-onset atrial fibrillation (AF). It was complicated by multiple arterial embolisms involving different organs despite the use of an intermediate dose of low-molecular-weight heparin (LMWH), and the patient was eventually discharged home on a direct-acting oral anticoagulant (DOAC).

## Introduction

The coronavirus severe acute respiratory syndrome coronavirus 2, SARS-CoV-2, responsible for the coronavirus disease-2019 (COVID-19) has rapidly spread, causing a global pandemic. The most-reported manifestations of the disease are respiratory symptoms such as fever, cough, sputum production, and shortness of breath. Also, upper airway symptoms like sore throat, nasal congestion, and rhinorrhea are perceived in patients presenting with the mild disease [1]. Nevertheless, extra-pulmonary manifestations were documented, such as cardiovascular complications, including acute myocardial injury, arrhythmia, acute pericarditis, left ventricular dysfunction, acute coronary syndrome leading to venous and arterial thromboembolic events [2]. Currently, various recommendations to prevent these events have emerged. However, thrombotic events might be the initial presenting symptoms of COVID-19 infected individuals [3].

## Case presentation

A 50-year-old man presented to the ED in late May 2020 with sudden onset excruciating pain on the posterior aspect of the right lower leg for two hours. The patient described a history of runny nose, sore throat, and noticed some palpitations over the previous five days. The patient had no reported past medical or surgical history. The patient was a nonsmoker, not an alcoholic with no history of illicit drug abuse. Family history was nonsignificant for a hereditable condition or vascular abnormalities. Initial vital signs were significant for a temperature of 98.8°F, a blood pressure of 129/76 mmHg, a heart rate of 78 beats/min, a respiratory rate of 18 cycles/min, and a body mass index of 31 kg/m2. On pulse oximetry, the patient was maintaining oxygen saturation on room air at 96%.

**Figure 1:**
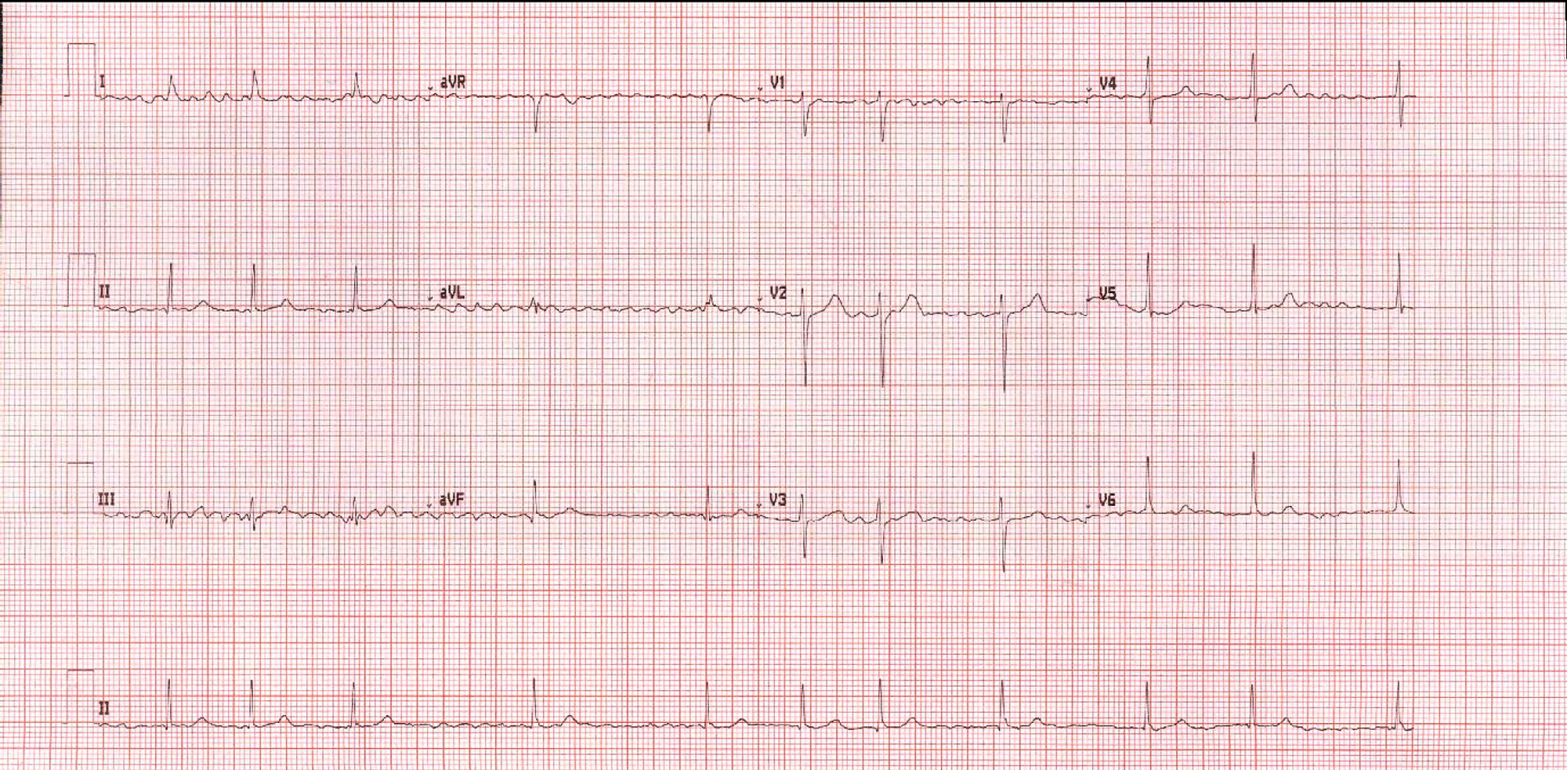
A 12-lead electrocardiogram showing AF with a pulse rate of 70 beats/min. AF, atrial fibrillation

On physical examination, the right posterior tibial artery and right dorsalis pedis artery were not palpable. Femoral and popliteal arteries were palpable bilaterally. The rest of the systemic examination is otherwise unremarkable. The 12 lead electrocardiogram (ECG) showed an irregular heart rate of 70 beats/min and was identified as new-onset atrial fibrillation (AF) (Figure 1). The cardiac enzymes were within the normal limit. Afterward, the patient had a real-time reverse transcriptase-polymerase chain reaction (RT-PCR) test of a nasopharyngeal swab specimen for SARS-CoV-2, and the result returned positive. Initial laboratory values showed a D-dimer level of 0.583 mcg/mL (Normal < 0.4 mcg/mL). Other laboratory results, including troponin T, thyroid-stimulating hormone value (TSH), complete blood count (CBC), comprehensive metabolic panel (CMP), and glycated hemoglobin (HbA1c) were unremarkable. A complete hypercoagulable workup, including measurements of factor V Leiden, protein C, protein S, and antithrombin III was unremarkable. 

**Figure 2:**
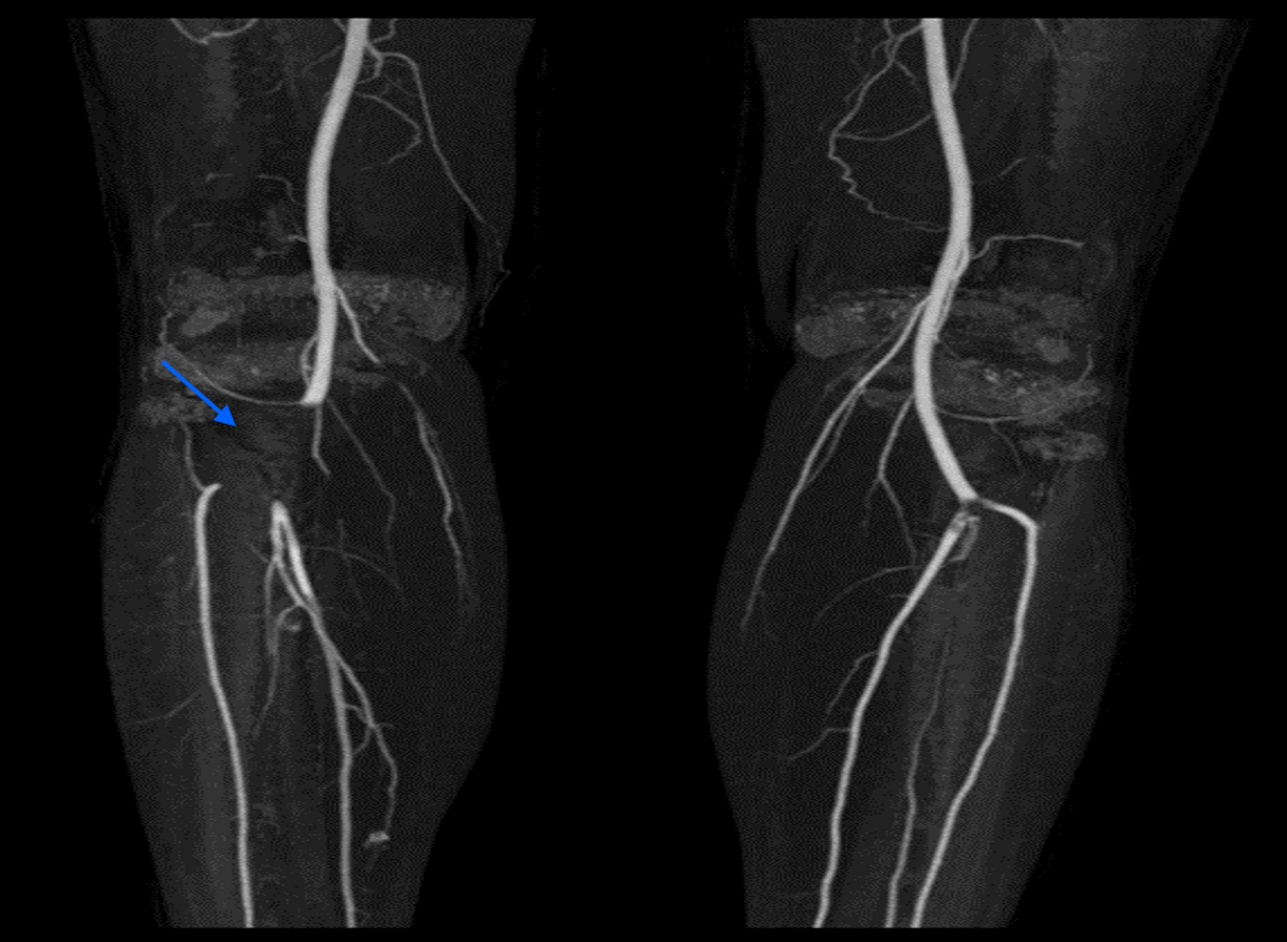
CT angiogram showing a partial right popliteal artery block.

Droplet and contact precautions ensued in addition to contact and eye protection. Symptomatic treatment with antipyretic paracetamol was initiated. Computed tomography angiography (CTA) of the lower extremity showed partial right popliteal artery block (Figure 2). Vascular surgery was involved, and the patient underwent catheter-directed therapy, including endovascular clot extraction to the right popliteal artery. The patient also initiated a therapeutic dose of enoxaparin of 1 mg/kg twice daily and oral warfarin with an international normalized ratio (INR) target between 2 and 3. The symptoms gradually improved and there was a notable improvement in his right posterior tibial and dorsal pedal pulses. He spent three days in the surgical intensive care unit (SICU) for observation. The patient received the SARS-CoV-2 treatment protocol of the hospital at that time. Azithromycin, oseltamivir, paracetamol, vitamin C, zinc sulfate, and hydroxychloroquine were given in addition to bisoprolol and omeprazole. A transthoracic echocardiogram (TTE) showed a normal size of the left ventricle and normal global systolic left ventricular function (ejection fraction of 55%) with no regional wall motion abnormality. The pressure and size of the right and left atrium were normal with no valvular abnormalities.

**Figure 3:**
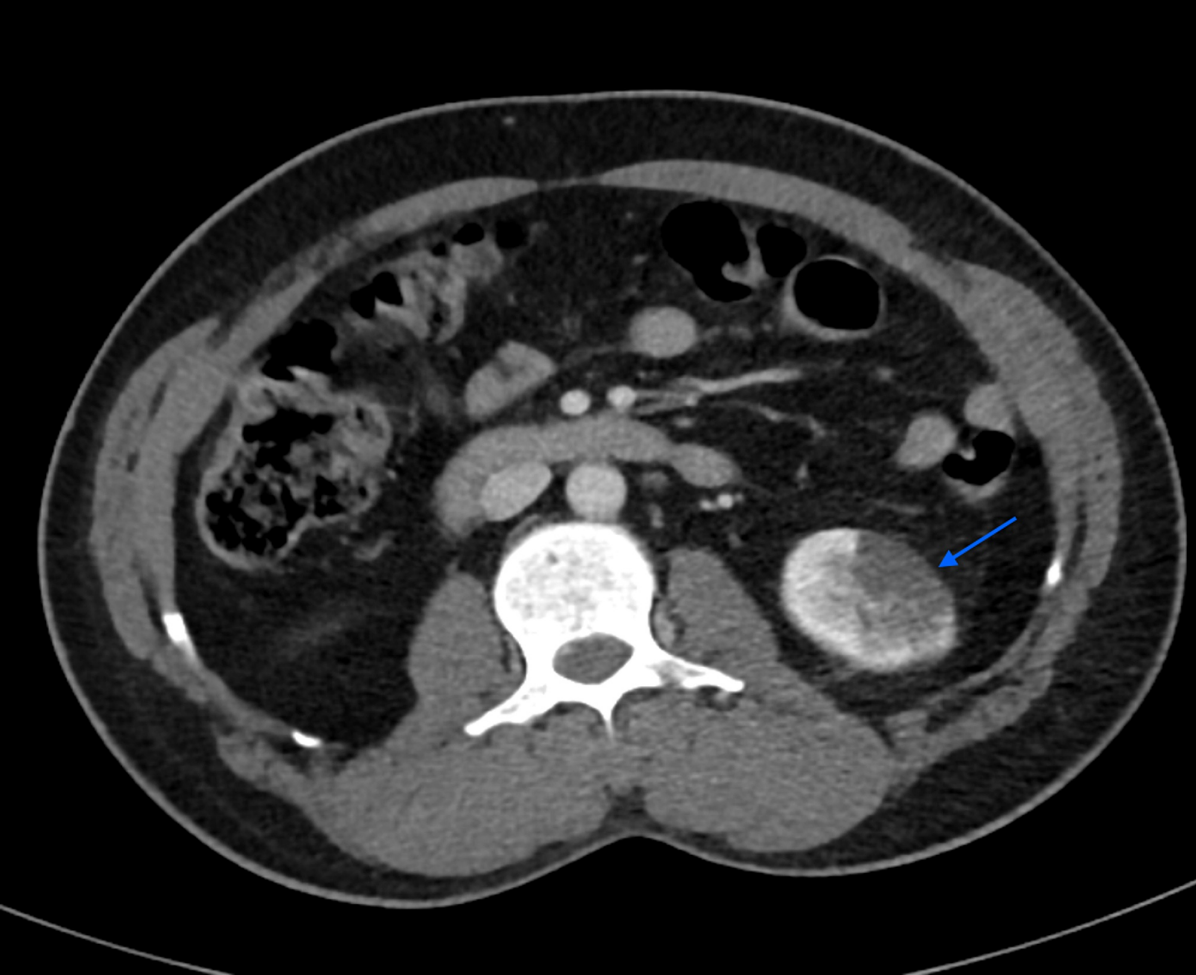
A wedge-shaped parenchymal defect at the inferior lobe of the left kidney.

**Figure 4:**
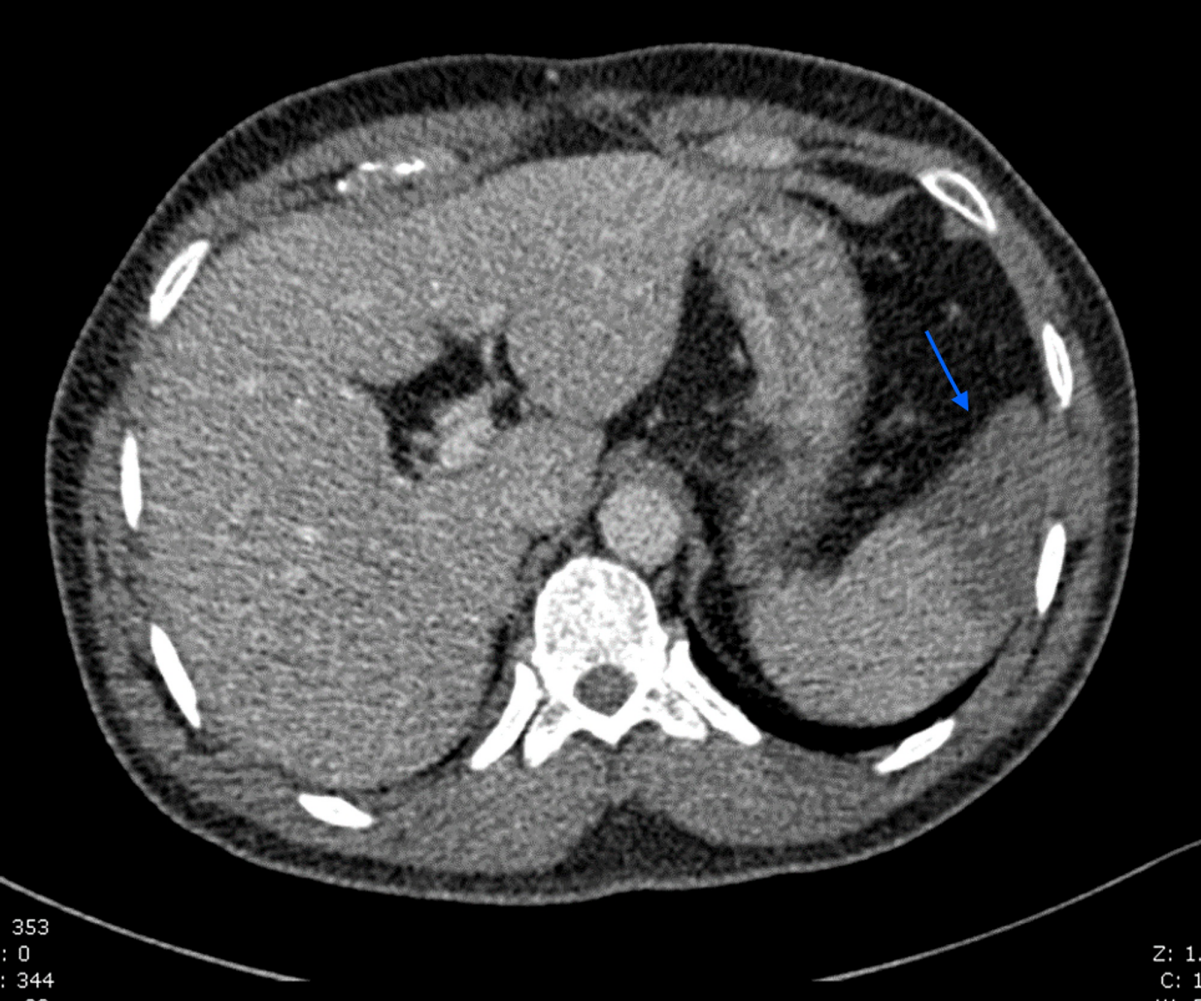
Large wedge-shaped hypoattenuating area involving the anterior and inferior aspects and partially extended to the hilum of the spleen.

In SICU, on the third day while the patient was under observation, he developed a dull abdominal pain mainly on the left side that required IV morphine. His abdomen was soft, nondistended, with no guarding or rebound tenderness and no organomegaly noticed on palpation. The CTA of the abdomen and pelvis with IV contrast revealed left kidney lower pole infarction (Figure 3) and high-grade narrowing of the anterior splenic artery thrombosis with multiple splenic infarctions involving the anterior and inferior aspects (Figure 4). The decision was to put the patient under observation for any concurrent complications and to be treated accordingly.

**Figure 5:**
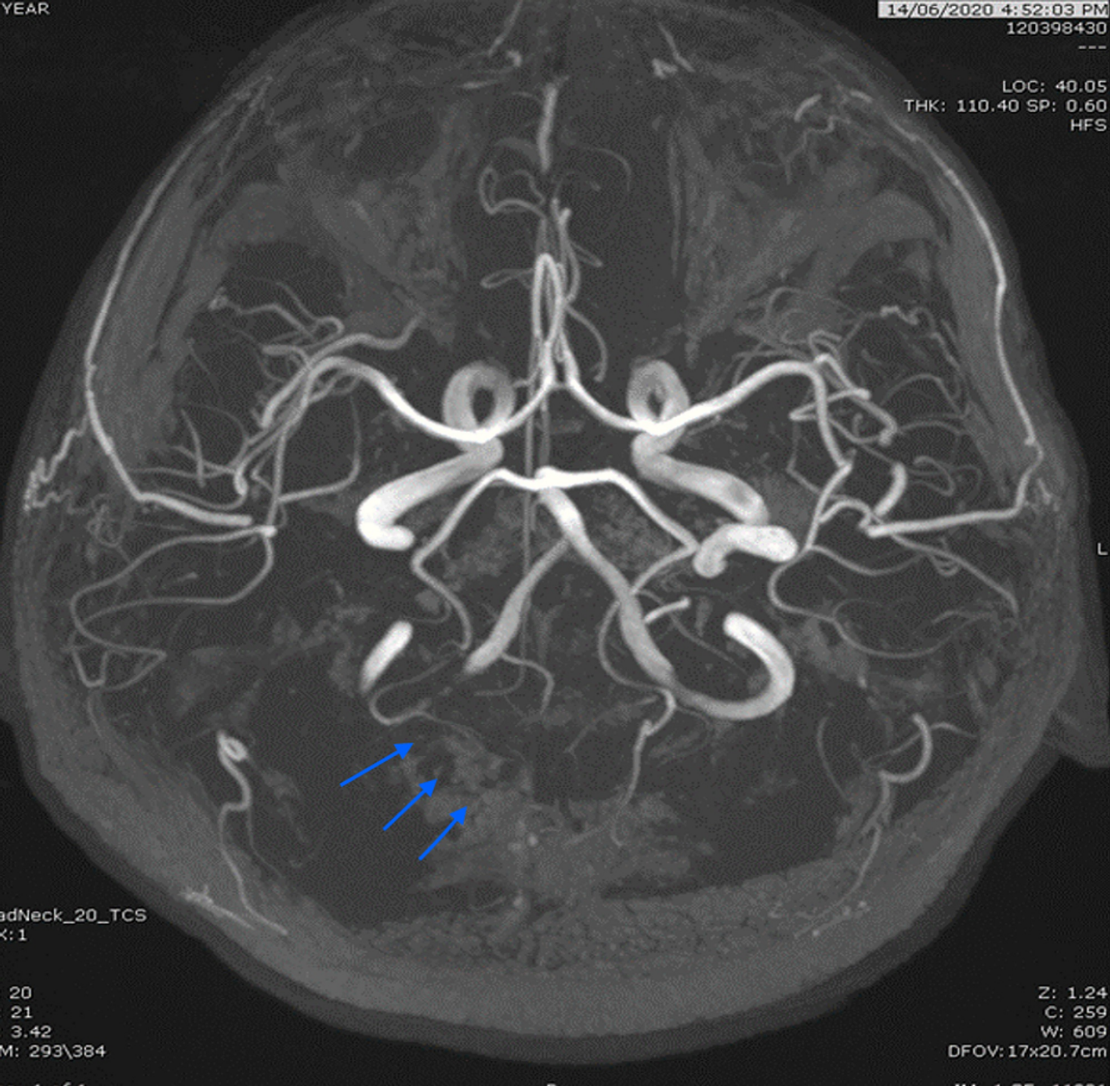
3D TOF MRA showing attenuated/occluded distal right PCA. 3D, three-dimensional; TOF, time-of-flight; MRA, magnetic resonance angiography; PCA, posterior cerebral artery

Over the course of the next week, the abdominal pain resolved, and the patient was transferred to the SARS-CoV-2 facility for quarantine. Five days later, he developed a symptom of blurry vision and headache, the patient denied having any similar symptoms before or had any eye discomfort. Focused neurological examination detected left homonymous hemianopia. Otherwise, it was unremarkable. Afterward, the ophthalmology team evaluated the patient and ordered magnetic resonance angiography (MRA) of the head. The latter showed the right occipital lobe subacute infarcts (Figure 5). The stroke team decided to continue anticoagulation with an INR target of 2-3, with a consultation to physical and occupational therapy. 

Four days later, a second RT-PCR nasopharyngeal swab was taken, which came back negative. The D-dimer level during the hospital stay continued to elevate, the last upon discharge was 4.74 mcg/mL. He was discharged home with an instruction to maintain direct-acting oral anticoagulant (DOAC), atorvastatin, aspirin, and bisoprolol with a close outpatient follow-up plan to an AF clinic for possible electrical cardioversion and a referral to a neuro-ophthalmologist clinic for rehabilitation therapy. On follow up, a transesophageal echocardiogram with saline study has been done which was normal with no cardiac shunts, intracardiac mural thrombus, and cardiac tumors. The patient was evaluated in the follow-up clinic with no vascular complaints or deterioration in his symptoms.

## Discussion

COVID-19 is caused by SARS-CoV-2, an enveloped beta coronavirus. Our knowledge regarding the pathophysiology of SARS-CoV-2 infection is still evolving. SARS-CoV-2 primarily affects the respiratory system [4]. However, several reports described cardiovascular complications [4]. These have been reported in multiple studies to be associated with hypercoagulability, increased risk for venous, and arterial thromboembolic events [5]. Studies show a variety in the prevalence of cardiovascular manifestations among patients with COVID-19 infection. In one study, Gopinathannair et al. reported the percentages of cardiac events within hospitalized patients infected with COVID-19. In a total of 1197 patients; AF occurred in 21%, acute pericarditis in 6.5%, pericardial effusion in 10.4%, sinus bradycardia in 8%, atrial flutter in 5.4%, 5.7% developed paroxysmal supraventricular tachycardia, nonsustained ventricular tachycardia (VT) in 6.3%, sustained monomorphic VT was reported by 3.8%, Torsade de Pointes by 3.5%, ventricular fibrillation by 4.8%, and pulseless electrical activity by 5.6% [6]. Several theories suggested explaining the transient hypercoagulable state including endothelial tissue injury through direct virus invasion, or indirectly by complement-mediated inflammatory response through acute phase reactants followed by cytokine storm (Interleukin 6, Interleukin 2, Interleukin 7, and tumor necrosis factor-α), stasis, and various other factors such as hypoxia, electrolyte disturbances, disseminated intravascular coagulation, drug-drug interaction, and lastly, positive antiphospholipid antibody [5]. Several viral infections such as influenza, H5N1, H1N1, parvovirus, cytomegalovirus (CMV), and Epstein-Barr virus (EBV) have been associated with thromboembolism [7].

As COVID-19 patients display a higher risk for VTE events, the American Society of Hematology recommends that all hospitalized patients should receive thromboprophylaxis in the form of low-molecular-weight heparin (LMWH) (preferred) or fondaparinux unless contraindicated. Also, they recommend that seriously ill/critical patients should receive a therapeutic dose of anticoagulant even in the absence of VTE. Upon discharge, patients switched into DOAC or vitamin K antagonist (warfarin). However, the data are still limited and needs further investigation [8].

Our patient also developed new-onset AF. Several proposed mechanisms include a reduction in angiotensin-converting enzyme 2 (ACE2) receptor availability, CD147- and sialic acid-spike protein interaction, enhanced inflammatory signaling eventually lead to inflammatory cytokine storm, sympathetic nervous system overactivity, direct viral endothelial damage, electrolytes disturbances, drug interaction, and acid-base balance disturbances in the acute phase of the disease [9]. Also, there is a historical relationship between viral infections and arrhythmia in patients with SARS‐CoV-1, MERS, and H1N1 [9]. The patient was relatively young with no known past medical history which could correlate to his hypercoagulable status and new-onset AF. This makes us think that there might be a relation between SARS-CoV-2 with thromboembolic events and AF supported by the elevated D-dimer level through the course of illness. Also, several cases have been reported as emboli are SARS-CoV-2-infected patients. However, the unique feature in our patient is the multiple systemic emboli which affected several organs system despite the use of LMWH.

## Conclusions

Evaluating patients with SARS-CoV-2 requires a high index of suspicions for any possible cardiovascular complications. Physicians should always be aware of these frequent complications. The pathophysiology of myocardial injury and arrhythmias in patients infected with SARS-CoV-2 is still vivid and needs further studies. We also thought that the D-dimer level could be a predictor for possible thromboembolic events.
